# Rumination and behavioural factors in Parkinson's disease depression

**DOI:** 10.1016/j.jpsychores.2016.01.008

**Published:** 2016-03

**Authors:** Camille L. Julien, Katharine A. Rimes, Richard G. Brown

**Affiliations:** a King's College London, Institute of Psychiatry Psychology and Neuroscience, Department of Psychology, London, UK; b Department of Health and Rehabilitation Psychology, Barts Health NHS Trust, London, UK

**Keywords:** Avoidance, Behaviour, CBT, Depression, Parkinson's disease, Rumination

## Abstract

**Objective:**

Parkinson's disease is associated with high rates of depression. There is growing interest in non-pharmacological management including psychological approaches such as Cognitive Behaviour Therapy. To date, little research has investigated whether processes that underpin cognitive models of depression, on which such treatment is based, apply in patients with Parkinson's disease. The study aimed to investigate the contribution of core psychological factors to the presence and degree of depressive symptoms.

**Methods:**

104 participants completed questionnaires measuring mood, motor disability and core psychological variables, including maladaptive assumptions, rumination, cognitive-behavioural avoidance, illness representations and cognitive-behavioural responses to symptoms.

**Results:**

Regression analyses revealed that a small number of psychological factors accounted for the majority of depression variance, over and above that explained by overall disability. Participants reporting high levels of rumination, avoidance and symptom focusing experienced more severe depressive symptoms. In contrast, pervasive negative dysfunctional beliefs did not independently contribute to depression variance.

**Conclusion:**

Specific cognitive (rumination and symptom focusing) and behavioural (avoidance) processes may be key psychological markers of depression in Parkinson's disease and therefore offer important targets for tailored psychological interventions.

## Highlights

• Depression severity in PD is associated with a range of psychological processes.
• The strongest associations with depression are with rumination, symptom focusing and avoidance.
• In contrast dysfunctional beliefs were not independently associated with depression.
• This suggests the potential to develop targeted psychological treatment.

## Introduction

Depression is common in Parkinson's disease, with over one third having a depressive disorder, subsyndromal depressive symptomatology or significant psychological distress [1]. There has been a welcome advance in the number and quality of randomised controlled trials (RCTs) of antidepressants, although a recent meta-analysis identified only small to moderate effect sizes [2], and the non- and partial-response rates to antidepressant medication are typically high in older adults and those with chronic physical health problems [3]. Management is further constrained by the potential for adverse drug interactions [4], evidence for suboptimal treatment by clinicians [5] and the facts that patients themselves express concerns about antidepressant medication [6], [7] with poor treatment adherence [8].

Explanations for depression in Parkinson's disease have typically focussed on potential biological based mechanisms with disturbances of brain monoamine systems [9]. However, the response to pharmacological treatments, and other evidence suggests a broader bio-psychosocial formulation is needed, both to define factors that may cause and maintain depression in the context of a progressive and disabling condition, and to guide the development of effective management [10]. Evidence supports an association between psychological and social factors and the presence and severity of depression in Parkinson's disease. For example, cognitive and behavioural avoidance as a coping response to illness related stressors, and negative illness representations are associated with depression, while active task-oriented coping tends to be associated with greater psychological well-being [11], [12], [13], [14]. While not necessarily causal, such factors may alter the risk of depression onset and relapse or serve to maintain symptoms. Such evidence has stimulated exploration of psychological approaches, particularly Cognitive Behaviour Therapy (CBT), as a potential alternative or adjunct to pharmacotherapy. Trial evidence [15], [16] suggests that significant treatment effects can be obtained [17], while the flexibility for delivering treatment in groups, by telephone [18] or via the internet, offers opportunity to reach a large number of patients, economically and in a way that some may prefer [6].

While evidence suggests that CBT can be effective, there are challenges where mood problems exist in the context of an objectively progressive and disabling condition with complex morbidity. Negative and unhelpful beliefs and assumptions, are central to the Cognitive Model of depression and a key treatment target [19]. However, the nature and content of such thoughts may be realistic in a chronic disease, and it is unknown whether they form a logical or useful therapeutic target in Parkinson's disease. Other potential targets may include maladaptive behavioural coping, unhelpful illness representations and repetitive thought processes such as worry and rumination [20], [21], [22], [23]. All of these may serve as vulnerability factors, and/or become activated in the context low mood to help maintain a depressive episode. There is the potential to modify CBT so that it targets those processes that contribute most to depression onset, maintenance and/or relapse, while reducing emphasis and time spent on less relevant elements of therapy.

The present study aimed to assess the relative contribution of a range of psychological factors either known to be associated with depression in Parkinson's disease, or predicted by the Cognitive Model that underpins conventional CBT. The results may support the value of current CBT approaches or suggest alternatives or adaptations with the potential to improve outcome.

## Methods

### Participants

Participants with a clinical diagnosis of Parkinson's disease [24] were recruited via an existing research cohort (‘Prospective Study of Mood States in Parkinson's Disease’ (PROMS-PD), Ethics Ref: 07/MRE01/9, UKCRN ID: 2519) [25]. The full cohort of 513 represented a consecutive series of consenting participants recruited over a 12 month period from specialist Parkinson's disease or Movement Disorder clinics in a number of centres in England and Wales. They had a mean age of 67.9 years (SD = 10.3, Range = 32–94 years) and 65% were male. Mean duration of PD was 6.9 years (SD = 6.0, Range = 0–39 years). Significant cognitive impairment (MMSE ≤ 24) [26] was present in 10.2%. Almost all participants in the cohort (94.9%) were taking antiparkinsonian medication at the time of recruitment.

From this cohort, participants for the present study were excluded if they had no given consent to be re-contacted about other studies; if they had a history of psychiatric disorder other than mood or anxiety disorder; a history of major neurological disorder other than Parkinson's disease (e.g. stroke), or a score of 24 or less on the MMSE [26]. Additionally, cohort participants who had been assessed as part of the main PROMS-PD study or other supplementary study within the previous 3 months were not approached to avoid over-testing. Written informed consent was obtained from all participants. Ethical approval for the study was granted by the South London Research Ethics Committee 4 (Reference 10/H0807/60).

### Assessment

Consenting participants completed a set of postal questionnaires assessing demographic and disease-related information, mood and psychological factors.

General physical health was measured using the physical health subscale from the Duke Older Americans Resources and Services (OARS) Multidimensional Functional Assessment Questionnaire (OMFAQ) [27]. Parkinson's disease related disability was assessed as a proxy for total motor and non-motor symptom burden using the Parkinson's Activities of Daily Living Scale (PADLS). The scale has been shown to correlate with clinical ratings of disease severity (r = 0.68) and duration (r = 0.39) [28].

Self-reported depressive symptoms were assessed using the Beck Depression Inventory Second Edition (BDI-II) [29], with a score of ≥ 14 indicating significant symptoms [30], [31]. A set of psychological factors were assessed. These were selected to cover a range of psychological constructs relevant to models of depression in general and in the context of a physical health condition. Where appropriate, for some measures, minor changes were made to the wording of the instructions and scale items to make them appropriate to older adults with PD.

1. The 24-item Dysfunctional Attitude Scale (DAS-24) [32] a self-report measure of general (rather than situation specific) unhelpful beliefs that an individual may hold about themselves, the outside world or their future, relating to aspects of personal achievement, dependency on others and self-control (e.g. ‘My happiness depends more on other people than it does on me’, ‘If I do not do as well as other people, it means I am an inferior human being’).
2. The Ruminative Responses Scale (RRS) from the Response Styles Questionnaire [33] is a 22-item scale, which assesses the general tendency to respond to negative emotions with ruminative thoughts, e.g. going somewhere to be alone and think about your feelings, dwelling or how sad you feel, or pondering vague questions such as ‘what am I doing to deserve this?’. Rumination is considered a metacognitive process (thinking about thinking and feelings) where the focus is the thinking style rather than the thought content.
3. The Cognitive and Behavioral Avoidance Scale (CBAS) [34] is a 31-item multi-dimensional measure of cognitive and behavioural avoidance, i.e. not thinking about or engaging in certain actions, or efforts to escape from situations, as a means of minimizing threat and distress. It has four reliable factors, Behavioral Social Avoidance (e.g. ‘I do not go out to events when I know there be a lot of people I do not know’), Behavioral Non-social Avoidance (e.g. I quit activities that challenge me too much), Cognitive Social Avoidance (e.g. ‘I just wait out tension in my relationships hoping that it will go away’) and Cognitive Non-social Avoidance (e.g. ‘I avoid making decisions about my future’).
4. The Revised Illness Perceptions Questionnaire (IPQ-R) [35] assesses dimensions of illness perceptions across a range of health conditions, focussed here on the participant's Parkinson's disease. The IPQ-R assesses aspects of the individual's personal beliefs and understanding through which they seek to make sense of their health condition: its nature and defining symptoms (identity), beliefs about cause(s), its timeline (acute or chronic), its consequences and long-term impact, and beliefs about control. These are assessed through a series of subscales. Following previous research on the instrument's properties in Parkinson's disease [36], four disease related symptoms were added to the existing checklist on the Identity subscale. An Emotional Representations subscale in the original was not used as it was judged to be very close conceptually to depression and might therefore bias data interpretation.
5. The Cognitive and Behavioural Responses to Symptoms Questionnaire (CBSQ) [37] is a measure of beliefs and behavioural responses to symptoms of their health condition. The form used measured five cognitive (belief) subscales: Fear Avoidance Beliefs (‘I am afraid that I will make my symptoms worse if I exercise’), Catastrophizing Beliefs (‘I worry that I may become permanently bedridden because of my symptoms’; Damage Beliefs (e.g. ‘symptoms are a signal that I am damaging myself’), Embarrassment Avoidance Beliefs (e.g. ‘the embarrassing nature of my symptoms prevents me from doing things’), and Symptom Focusing, (e.g. ‘I think a great deal about my symptoms’). Two behavioural subscales assessed ‘All or Nothing’ Behaviour (e.g. ‘I tend to overdo things and then rest up for awhile’) and Avoidance/Resting (e.g. ‘when I experience symptoms, I rest’).

### Statistical analyses

All analyses were performed using SPSS version 17.0 for Windows (SPSS Inc.). Data were screened for outliers and missing values. No transformations were judged necessary and all variables met criteria for the assumptions of multivariate analysis [38].

Hierarchical multiple regression analyses were planned to determine the proportion of variance in the current depression (BDI-II) accounted for by psychological variables, in addition to that explained by the planned covariate of motor disability (PADLS). In order to reduce the number of independent variables entered into the main regression model, subscales of the IPQ-R and CBSQ were entered into separate multiple regression analyses to identify the strongest predictors of depression symptom severity, as measured by the BDI-II (dependent variable). Given the multiple variables, alpha level of the final model was set conservatively to 0.01.

Separately, logistic regression was planned to determine the strongest predictors of depression status (BDI-II score ≥ 14). Predictors were transformed into dichotomous variables, using their median as the cut-off score. Although less sensitive than using continuous scores, the findings provide accessible summaries of potential important predictors of outcome, even if confidence intervals tend to be large.

## Results

### Participant characteristics

Of 173 patients invited, 104 returned completed analysable questionnaires. Of those not included, 15 declined to participate and 48 did not respond. A further 11 participants were subsequently excluded, five because the reported another major neurological disorders and six because they returned incomplete datasets. Demographic and clinical information for the total sample is summarised in Table 1 together with the characteristics of the two subgroups. The ‘depressed’ subgroups had a significantly younger age of onset of the PD (t(103) = 1.59, p = 0.006) and longer disease duration (t(103) = 2.87, p = 0.005). The self-reported disability (PADLS) was higher in the ‘depressed’ group (Fishers Exact Test, probability = 0.003_,_ Chi-square (df = 3) = 13.31, p = 0.004). Moderate or severe functional difficulties were reported by 54% of the ‘depressed’ subgroup compared to only 29.1% of the ‘non-depressed’ subgroup. The subgroups did not differ significantly in terms of age, gender or frequency of co-morbid physical health conditions. Details of depression and other psychological variables are provided in Table 2. In total, 49.3% reported symptoms of depression (BDI-II ≥ 14) with 26.9% in the moderate to severe range (BDI-II ≥ 20). The ‘depressed’ subgroups scored significantly higher (t(103) > 2.70, p < 0.01) on all scales and subscales with the exception of IPQ-R Identity, Illness Coherence and Control subscales, and CBSQ Fear Avoidance subscale.

### Preliminary linear regression analyses

The IPQ-R explained 31.2% of the variance in depression (*R*^2^ = 0.31, F(11, 92) = 3.79, p < .001). Of the subscales, Consequences emerged as a significant predictors of depression and was retained for the main analysis. The CBSQ explained 48.5% of the variance in depression (*R*^2^ = 0.49, F(5, 98) = 18.49, p < .001). Symptom Focusing and Avoidance/Resting emerged as significant predictors of depression severity and both were retained. Table 3 shows the correlations matrix between the BDI-II and the set of independent variables retained for inclusion in the main regression analysis. All independent variables had inter-correlation coefficients of less than 0.66 (i.e. less than 44% shared variance).

### Main linear regression analysis: psychological predictors of depression

Table 4 displays the results of the main regression model for psychological factors and disability on depression. The adjusted *R*^2^ (= 0.708) indicates that the variables in the final regression equation explained almost 71% of the variability in depression symptom severity (*R*^2^ = 0.73, F(6, 97) = 42.6, p < .001), with psychological factors contributing 58.9% to the variance. Rumination (RSS), Cognitive-behavioral Avoidance (CBAS Total), and Avoidance/Resting and Symptom Focusing (CBSQ) emerged as significant predictors, but not dysfunctional attitudes and beliefs (DAS-24). A secondary analysis of the CBAS showed that Behavioral Social Avoidance was the only subscale to significantly contribute to depression variance.

Table 5 displays the results of the logistic regression analysis determining the relative contribution of psychological factors to the dichotomous risk of being depressed (BDI-II ≥ 14), beyond that attributable to differences in disability. Adjusted odds ratios (Exp B) and their 95% confidence intervals are presented for all variables, as well as Hosmer and Lemeshow's *R*^2^ and the chi-square statistic for the final model. Significance was determined using the Wald statistic. The final model correctly classified 86.5% of cases (*χ*^2^ = 85.99, p < .001), with Hosmer and Lemeshow's *R*^2^ value indicating that the model explained 59.7% of the variance in depression outcome. The Hosmer and Lemeshow's goodness-of-fit test (*χ*^2^ = 10.18, p = .253) indicated that the final regression model fitted the observed data. Significant predictors of depression in the final model were high rumination, high cognitive-behavioural avoidance, high symptom focusing, high avoidance/resting and ‘all-or-nothing’ behaviour, although the confidence intervals were all large. Individuals who scored highly (total score > 13) on rumination were approximately 9.4 times (CI 2.26–39.31) more likely to be depressed than low ruminators. High avoiders (total score > 56) were approximately 15.0 times (CI 3.07–71.00) more likely than low avoiders to be depressed, and individuals who frequently focused on their Parkinson's symptoms (total score > 8) were approximately 6.0 times (CI 1.57–25.57) more likely than individuals who focused on their symptoms less frequently.

## Discussion

This study examined the potential contributions of a range of psychological factors to depression in Parkinson's disease, beyond that explained by motor disability. Findings showed that psychological factors account for a substantial proportion of variance in outcome.

The finding that cognitive and behavioural avoidance emerged as a strong correlate of depression is consistent with many previous studies in Parkinson's disease [11], [12], [20], [39]. In particular, behavioural avoidance of social activities was significantly predictive, above other forms of avoidance. Whether because of concerns such as falling in public or fear of negative social evaluation concerning their Parkinson's symptoms, patients often avoid social situations. The initial short-term reduction of stress and anxiety related to such situations can then develop over time into a more chronic pattern of avoidance. The resultant isolation and loss of opportunity for rewarding interactions may provide a plausible mechanism for vulnerability to depression and maintaining depressed mood once it occurs. Behavioural interventions within CBT approaches for depression target such avoidant coping and seek to reintroduce a range of social activity with associated benefits for mood. The present evidence supports such as strategy.

Novel findings in the present study were evidence of associations between depression, rumination and symptom focusing in PD. Along with avoidance, they emerged as strongly associated with depression symptom severity and depression status. Rumination is a trait-like self-reflective cognitive mode that involves ‘repetitively and passively focusing one's symptoms of distress and on the possible causes and consequences’ [40]. Rumination increases in depression, is associated with the severity and duration of depressive episodes [41], and increases the risk of depressive relapse in remitted patients [42], suggesting it may be an important vulnerability factor. Moreover, once depressed, rumination may serve to maintain negative affect through the repetitive focus on negative events, problems and their implications in the perceived absence of a solution. Rumination may develop as a preferred way of coping with challenges and changing circumstances, particularly for individuals who have difficulty tolerating uncertainty and relinquishing or reappraising important life goals. The sense of certainty and relief from responsibility that follows an appraisal of a situation as hopeless may in turn reinforce the use of rumination as a coping strategy. This might subsequently exacerbates pessimistic thinking and depressed mood, while inhibiting more active problems solving [43]. Metacognitive beliefs about the perceived positive value of rumination and other strategies haven been shown to occur in Parkinson's disease and be associated with psychological distress [44], [45].

The role of rumination as a psychological predictor of depression in Parkinson's disease is also particularly relevant in light of its known, and possibly reciprocal, association with attention and executive function [46], [47]. Such cognitive deficits, common in Parkinson's disease [48], may exacerbate individuals' tendency to ruminate by further inhibiting their ability to switch their attention from such think patterns to more adaptive problem-solving strategies. However, there is no evidence that the severity of executive dysfunction is predictive of response to CBT in Parkinson's disease [49]. Neuroimaging evidence in psychiatric patients with depressive disorder also points to abnormal processing and network interactions involving anterior cingulate and orbitofrontal cortex during rumination and self-referential processing [50]. Pathophysiological changes in these same regions in Parkinson's disease may predispose some patients to rumination and increase risk of depression, even in the absence of cognitive impairment.

Symptom focusing (inwardly directed attention to physical symptoms of disease) was also associated with depression severity and depression status, consistent with another recent report examining anxiety and distress in Parkinson's disease [45]. Symptom focusing has predominantly been studied in relation to health anxiety, where it is thought to play a key maintaining role by increasing preoccupation with bodily symptoms and perceptions of illness threat [51]. Symptom focusing also correlated strongly here with cognitive-behavioural avoidance and avoidance/resting, consistent with a hypothesis that attentional somatic focus may lead to attempts to manage symptoms by avoiding activity and/or engaging in excessive resting, and accompanying positive beliefs about the value of such strategies.

Contrary to expectations, although dysfunctional assumptions were associated with depressed mood, the effect size was small compared to other factors studied (Table 3), and did not make a significant independent contribution to explaining depression in multivariate analyses. It is possible that dysfunctional assumptions play a more important role in the initial onset of depression, which was not assessed in the current study, while other processes, such as rumination and avoidance, are more instrumental in maintaining and exacerbating depressive symptoms. The present finding is also consistent with the suggestion that it is not the level of unhelpful beliefs that is key in the onset of depression but rather the ease with which these beliefs are reactivated by low mood, sometimes known as ‘cognitive reactivity’ [52].

The present study has implications for how future CBT interventions might be tailored to the specific needs of people with Parkinson's disease, targeting potentially key maladaptive processes of rumination, symptom focusing and cognitive-behavioural avoidance, with less emphasis placed on cognitive distortions or dysfunctional beliefs or assumptions. Such targeting may also offer the promise of briefer yet still effective interventions. A range of possible ‘third-wave’ cognitive therapies are emerging, designed specifically to target rumination and related metacognitive processes, particularly when depression is persistent or recurrent. These include Rumination-focused CBT (RFCBT) [53] Mindfulness-based CBT (MBCT) [54] and Metacognitive Therapy [55]. Group MBCT has been piloted in Parkinson's disease and a qualitative study found the intervention to be beneficial, particularly in relation to developing and consolidating coping skills in the context of actual loss, and perceived stigma than can drive avoidance [56].

Some limitations of the present study should be acknowledged. As a postal survey, even with reasonable response rates, there is the potential for selection bias. Future research should seek to recruit from a consecutive clinic population, ideally with face-to-face assessment. As the study only included participants with a score above 24 on the MMSE, results may not be generalizable to depressed patients with significant cognitive impairment. The cross-sectional nature of the study means that caution is necessary in inferring causality from the patterns of association. Intervention studies that target specific components would provide insights in the causal nature of the relationships as well as inform clinical management.

In conclusion, the present study reinforces the important contribution of psychological factors to depression in Parkinson's disease. It further indicates that clinical interventions that primarily target behavioural, cognitive and metacognitive processes, including avoidance, symptom focusing and rumination offer the potential to reduce depressive symptoms or depression risk.

## Conflict of interest statement

The authors have no competing interests to report.

## Figures and Tables

**Table 1:** Demographic and clinical characteristics (n = 104).

Variable	Total sample (N = 104)		‘Non-depressed’ (BDI-II < 14) (N = 53)		‘Depressed’ (BDI-II ≥ 14) (N = 51)	
Mean (SD) or %	Range	Mean (SD) or %	Range	Mean (SD) or %	Range
Age (years)	67.8 (8.5)	49–87	68.9 (8.4)	52–87	66.3 (8.4)	49–83
Gender (% male)	60	–	60	–	58	–
Age at PD onset (years)	58.1 (10.2)	35–79	60.8 (8.8)	39–75	55.2 (10.8)	35–79
Duration of PD (years since diagnosis)	10.0 (5.6)	3–28	8.4 (4.7)	1–24	11.5 (6.2)	4–28
Disability (PADLS) (%)						
No difficulties	10.6	–	16.4	–	4.0	–
Mild difficulties	48.1	–	54.5	–	42.0	–
Moderate difficulties	30.8	–	27.3	–	34.0	–
Severe difficulties	10.6	–	1.8	–	20.0	–
OARS symptom count	2.2 (1.8)	0–8	1.8 (1.4)	0–5	2.6 (2.0)	0–8

PD: Parkinson's disease; PADLS: Parkinson's Activities of Daily Living Scale; OARS: Duke Older Americans Resources and Services Questionnaire.

**Table 2:** Depression and psychological variables (n = 104).

Variable	Total sample (N = 104) Mean (SD)	‘Non-depressed’ (BDI-II < 14) (N = 53) Mean (SD)	‘Depressed’ (BDI-II ≥ 14) (N = 51) (Mean SD)
BDI-II total	14.1 (8.2)	7.9 (3.3)	20.8 (6.3)
DAS-24 total	85.7 (18.7)	79.8 (17.84)	92.2 (17.6)
RRS total	14.8 (11.2)	8.1 (7.2)	22.3 (10.1)
CBAS total	62.9 (22.2)	48.8 (12.0)	78.5 (20.4)
CBAS Behavioral Social scale	14.8 (7.0)	10.5 (3.5)	19.5 (6.1)
CBAS Behavioral Non-Social scale	13.9 (4.3)	11.4 (3.2)	16.7 (3.7)
CBAS Cognitive Social scale	13.2 (5.6)	10.2 (3.5)	16.6 (5.8)
CBAS Cognitive Non-Social scale	21.0 (7.5)	16.7 (4.9)	25.8 (7.0)

*IPQ-R*			
Identity scale	6.9 (3.2)	6.3 (3.0)	7.5 (3.2)
Consequences scale	22.6 (4.3)	21.2. (4.4)	24.2. (3.7)
Timeline scale	39.5 (4.4)	38.4 (4.1)	40.6 (4.4)
Control scale	34.8 (5.4)	35.6 (5.8)	33.9 (5.0)
Illness coherence scale	16.6 (4.5)	16.8 (4.2)	16.3 (4.8)

*CBSQ*			
Fear Avoidance scale	5.1 (3.6)	4.4 (3.4)	5.8 (3.8)
Embarrassment Avoidance scale	6.8 (4.0)	5.4 (3.7)	8.3 (3.7)
Symptom Focusing scale	7.3 (3.5)	5.5 (3.1)	9.2 (2.8)
All or Nothing scale	5.9 (3.7)	4.5 (3.4)	7.4 (3.5)
Avoidance/Resting scale	8.6 (5.2)	6.1 (4.0)	11.3 (4.9)

BDI-II: Beck Depression Inventory Second Edition; DAS-24: 24-item Dysfunctional Attitude Scale; CBAS: Cognitive and Behavioral Avoidance Scale; IPQ-R: Illness Perception Questionnaire — Revised; RSS: Ruminative Response Scale; CBSQ: Cognitive and Behavioral Responses to Symptoms Scale.

**Table 3:** Inter-correlations between dependent and independent variables selected for the main linear regression analyses.

	PADLS	DAS	CBAS	RRS	IPQ consequences	CBSQ symptom focus	CBSQ avoidance/resting.
BDI-II	.357⁎⁎	.344⁎⁎	.778⁎⁎	.724⁎⁎	.365⁎⁎	.510⁎⁎	.588⁎⁎
PADLS		.116	.409⁎⁎	.240⁎	.421⁎⁎	.148	.457⁎⁎
DAS-24 (total)			.345⁎⁎	.445⁎⁎	.050	.251⁎	.121
CBAS (total)				.656⁎⁎	.326⁎⁎	.381⁎⁎	.542⁎⁎
RRS (total)					.223	.499⁎⁎	.434⁎⁎
IPQ-R consequences						.291⁎⁎	.413⁎⁎
CBSQ symptom focusing							.368⁎⁎

BDI-II: Beck Depression Inventory Second Edition; PADLS: Parkinson's Activities of Daily Living Scale; DAS-24: 24-item Dysfunctional Attitude Scale; CBAS Cognitive and Behavioral Avoidance Scale; RRS: Ruminative Responses Scale; IPQ-R: Illness Perceptions Questionnaire Revised; CBSQ: Cognitive and Behavioral Responses to Symptoms Questionnaire.

⁎ p < .05.

⁎⁎ p < .01.

**Table 4:** Final cross-sectional multiple linear regression model of psychological predictors and disease severity on depression symptom severity (BDI-II).

	B	SE B	β
Constant	− 4.20	2.52	
Disease severity (PADLS)	0.10	0.61	.010
Dysfunctional assumptions (DAS-24 total)	0.004	0.03	.009
Rumination (RRS total)	0.21	0.06	.292⁎⁎
Cognitive-behavioral avoidance (CBAS total)	0.16	0.03	.437⁎⁎
Avoidance/Resting (CBSQ)	0.27	0.11	.171⁎
Symptom focusing (CBSQ)	0.31	0.15	.131⁎

PADLS: Parkinson's Activities of Daily Living Scale; DAS-24: 24-item Dysfunctional Attitude Scale; RRS: Ruminative Responses Scale; CBAS Cognitive and Behavioral Avoidance Scale; CBSQ: Cognitive and Behavioral Responses to Symptoms Questionnaire.

⁎ p < .05, *R*^2^ = 0.725. Adjusted *R*^2^ = 0.708.

⁎⁎ p < .01, *R*^2^ = 0.725. Adjusted *R*^2^ = 0.708.

**Table 5:** Final logistic regression model of psychological predictors and disease severity on depression status (BDI-II ≥ 14).

	Exp (B) (odds ratio)	95% C.I. for Exp (B)
Constant	0.004	
Motor disability (PADLS ≥ 2)	1.18	0.25–5.49
Dysfunctional assumptions (DAS-24 ≥ 85)	2.60	0.59–11.40
Rumination (RRS ≥ 13)	9.42**	2.26–39.31
Cognitive-behavioural avoidance (CBAS ≥ 56)	14.77**	3.07–71.00
Symptom focusing (CBSQ subscale ≥ 8)	6.33**	1.57–25.57
Avoidance/Resting (CBSQ subscale ≥ 9)	6.99*	1.50–32.60
All-or-nothing behaviour (CBSQ subscale ≥ 5)	4.74*	1.15–19.63

Note: *R*^2^ = .597; Model χ^2^ = 85.99, p < .001, *p < .05; **p < .01.

PADLS: Parkinson's Activities of Daily Living Scale; DAS-24: 24-item Dysfunctional Attitude Scale; RRS: Ruminative Responses Scale; CBAS Cognitive and Behavioral Avoidance Scale; CBSQ: Cognitive and Behavioral Responses to Symptoms Questionnaire.
